# Dental Treatment in Schools

**Published:** 1920-12-18

**Authors:** 


### Dental Treatment in Schools.

In a valuable contribution to the *British Journal of Dental Science*, Mr. J. E. Hogan has just given a careful account of the methods employed and the results observed in his capacity as dental officer in a small school clinic. The importance of his observations lies in the fact that the school was small enough for him to give careful and continuous individual treatment, and to notice the effect of that treatment on individual boys and girls, and therefore to draw sound general conclusions. Care of the teeth is now recognised to be one of the principal methods of preserving or improving the health of children attending school, for decayed teeth and septic mouths are a prolific cause of disease and debility in the earlier years of life.

Without going into the description of his technical methods, it is sufficiently suggestive to record that Mr. Hogan by systematic treatment was able in five years to bring about a transformation in the dental health of this small local school. In the first year, out of seventy-one children, aged from six to ten, only five had teeth free from all decay, and twenty-one free from decay of the permanent teeth. In 1919, out of 153 children, aged from six to fourteen, eighty-six were free from all decay, and 120 free from decay of the permanent teeth. Early and regular treatment, Mr. Hogan points out, means a minimum of dead teeth, which would do away with the habit now so prevalent of accusing dead teeth of being the parent of many systemic diseases of otherwise obscure or doubtful origin. Inspection and treatment cannot begin too early. The earliest age he got in this school was five years, but it is not nearly enough.

But apart from the inference to be drawn as to the value and importance of introducing a standardised method of treatment in the schools, the most interesting passages are those which deal with caries in relation to material prosperity and food. The large majority of those attending the school were the children of small farmers and labourers. These had much the best teeth. Their diet was mainly dry bread made from home-ground flour, with tea, as the morning meal; for the evening, boiled potatoes, and occasionally a very small quantity of boiled fat bacon. They had only a small quantity of sugar in their tea.

The minority consisted of the children of the household servants of Lord Gough, who were not natives, and usually came from the cities. These children's teeth were much worse than the others, their diet was very varied, and according to ordinary standards the best. "I have found as a general rule," declares Mr. Hogan, "that the more extensive dental caries was associated with material prosperity." Material prosperity and bad teeth are certainly a significant correlation which serves to emphasise the need of efficient school clinics.

